# ^18^F-FDG PET/CT oncologic imaging at extended injection-to-scan acquisition time intervals derived from a single-institution ^18^F-FDG-directed surgery experience: feasibility and quantification of ^18^F-FDG accumulation within ^18^F-FDG-avid lesions and background tissues

**DOI:** 10.1186/1471-2407-14-453

**Published:** 2014-06-19

**Authors:** Stephen P Povoski, Douglas A Murrey, Sabrina M Smith, Edward W Martin, Nathan C Hall

**Affiliations:** 1 Division of Surgical Oncology, Department of Surgery, Arthur G. James Cancer Hospital and Richard J. Solove Research Institute and Comprehensive Cancer Center, The Ohio State University Wexner Medical Center, Columbus, OH 43210, USA; 2 Division of Molecular Imaging and Nuclear Medicine, Department of Radiology, The Ohio State University Wexner Medical Center, Columbus, OH 43210, USA; 3 College of Medicine, The Ohio State University, Columbus, OH 43210, USA

**Keywords:** ^18^F-FDG, PET/CT, SUV_max_, Injection-to-scan acquisition time, Delayed imaging, Lesion-to-background ratio, Tumor-to-background ratio, ^18^F-FDG-directed surgery, Real-time, Oncologic

## Abstract

**Background:**

^18^F-fluorodeoxyglucose (^18^F-FDG) positron emission tomography/computed tomography (PET/CT) is a well-established imaging modality for a wide variety of solid malignancies. Currently, only limited data exists regarding the utility of PET/CT imaging at very extended injection-to-scan acquisition times. The current retrospective data analysis assessed the feasibility and quantification of diagnostic ^18^F-FDG PET/CT oncologic imaging at extended injection-to-scan acquisition time intervals.

**Methods:**

^18^F-FDG-avid lesions (not surgically manipulated or altered during ^18^F-FDG-directed surgery, and visualized both on preoperative and postoperative ^18^F-FDG PET/CT imaging) and corresponding background tissues were assessed for ^18^F-FDG accumulation on same-day preoperative and postoperative ^18^F-FDG PET/CT imaging. Multiple patient variables and ^18^F-FDG-avid lesion variables were examined.

**Results:**

For the 32 ^18^F-FDG-avid lesions making up the final ^18^F-FDG-avid lesion data set (from among 7 patients), the mean injection-to-scan times of the preoperative and postoperative ^18^F-FDG PET/CT scans were 73 (±3, 70-78) and 530 (±79, 413-739) minutes, respectively (P < 0.001). The preoperative and postoperative mean ^18^F-FDG-avid lesion SUV_max_ values were 7.7 (±4.0, 3.6-19.5) and 11.3 (±6.0, 4.1-29.2), respectively (P < 0.001). The preoperative and postoperative mean background SUV_max_ values were 2.3 (±0.6, 1.0-3.2) and 2.1 (±0.6, 1.0-3.3), respectively (P = 0.017). The preoperative and postoperative mean lesion-to-background SUV_max_ ratios were 3.7 (±2.3, 1.5-9.8) and 5.8 (±3.6, 1.6-16.2), respectively, (P < 0.001).

**Conclusions:**

^18^F-FDG PET/CT oncologic imaging can be successfully performed at extended injection-to-scan acquisition time intervals of up to approximately 5 half-lives for ^18^F-FDG while maintaining good/adequate diagnostic image quality. The resultant increase in the ^18^F-FDG-avid lesion SUV_max_ values, decreased background SUV_max_ values, and increased lesion-to-background SUV_max_ ratios seen from preoperative to postoperative ^18^F-FDG PET/CT imaging have great potential for allowing for the integrated, real-time use of ^18^F-FDG PET/CT imaging in conjunction with ^18^F-FDG-directed interventional radiology biopsy and ablation procedures and ^18^F-FDG-directed surgical procedures, as well as have far-reaching impact on potentially re-shaping future thinking regarding the “most optimal” injection-to-scan acquisition time interval for all routine diagnostic ^18^F-FDG PET/CT oncologic imaging.

## Background

^18^F-fluorodeoxyglucose (^18^F-FDG) positron emission tomography/computed tomography (PET/CT) is a well-established imaging modality for a wide variety of solid malignancies [1-5]. Its utilities have included initial cancer diagnostics, staging, restaging, therapy planning, therapy response monitoring, surveillance, and cancer screening for at-risk populations. Beyond these utilities, there has been growing interest in evaluating the feasibility of utilizing ^18^F-FDG and PET/CT technology for providing real-time information within the operative room and perioperative arena [6-62].

As part of an effort to provide surgeons with improved intraoperative tumor localization and image-based verification of completeness of resection, our collaborative group at The Ohio State University has previously described a novel, multimodal imaging and detection strategy involving perioperative patient and *ex vivo* surgical specimen ^18^F-FDG PET/CT imaging performed in combination with intraoperative ^18^F-FDG gamma detection [51]. As part of this schema, patients could undergo both a same-day preoperative diagnostic whole-body ^18^F-FDG PET/CT and a same-day postoperative diagnostic limited field-of-view ^18^F-FDG PET/CT, utilizing a single preoperative dose of ^18^F-FDG. This has provided our group with a unique dual-set of diagnostic ^18^F-FDG PET/CT images, in which the initial same-day preoperative diagnostic whole-body ^18^F-FDG PET/CT images were acquired within the injection-to-scan acquisition time interval generally recommended for diagnostic whole-body ^18^F-FDG PET/CT imaging [63], and in which the second set of same-day diagnostic limited field-of-view ^18^F-FDG PET/CT images were acquired after the completion of the surgical procedure, once the patient had completed standard postoperative recovery in the post-anesthesia care unit. This second set of same-day diagnostic limited field-of-view ^18^F-FDG PET/CT images was highly dependent upon the length of the surgical procedures performed, thus creating injection-to-scan acquisition time intervals for that second set of same-day diagnostic limited field-of-view ^18^F-FDG PET/CT images at time points far beyond what is generally described.

The current retrospective data analysis was undertaken to examine ^18^F-FDG-avid lesions and corresponding background tissues on same-day preoperative and postoperative ^18^F-FDG PET/CT scans to assess the feasibility and quantification of diagnostic ^18^F-FDG PET/CT oncologic imaging at extended injection-to-scan acquisition time intervals. Herein, we have: (1) demonstrated the ability to acquire diagnostic quality images at extended injection-to-scan acquisition times; (2) identified and quantified the amount of ^18^F-FDG accumulation in ^18^F-FDG-avid lesions and in corresponding background tissues at these extended injection-to-scan acquisition times; and (3) compared the amount of ^18^F-FDG accumulation in ^18^F-FDG-avid lesions and in corresponding background tissues at these extended injection-to-scan acquisition times to that of the corresponding injection-to-scan acquisition time interval generally recommended for diagnostic whole-body ^18^F-FDG PET/CT oncologic imaging.

## Methods

All aspects of the current retrospective analysis were approved by the Cancer Institutional Review Board (IRB) at The Ohio State University Wexner Medical Center. The data for the current retrospective analysis were acquired from a master prospectively-maintained database (with database inclusion dates from June 2005 to June 2012), which were generated from the combination of several Cancer IRB-approved protocols, and which involved a multimodal imaging and detection approach to ^18^F-FDG-directed surgery for the localization and resection of ^18^F-FDG-avid lesions in patients with known and suspected malignancies. Depending upon the clinical scenario, these ^18^F-FDG-directed surgical procedures were performed with either the intent for curative resection, for palliation, or for making a definitive tissue diagnosis, as based upon the standard of care management for any given disease presentation.

All patients who were eligible to be included in this current retrospective analysis consisted of those individuals who: (1) received a same-day single-dose preoperative intravenous injection of ^18^F-FDG; (2) underwent same-day preoperative diagnostic whole-body ^18^F-FDG PET/CT scan (usually consisting of 6 to 8 field-of-view PET bed positions, and with 2 minutes of PET imaging for each field-of-view PET bed position); (3) proceeded to the operating room for their anticipated surgical procedure and completed standard postoperative recovery in the post-anesthesia care unit; and (4) underwent a same-day postoperative diagnostic limited field-of-view ^18^F-FDG PET/CT scan (which was limited only to the immediate area of the surgical resection field, usually consisting of 1 to 3 field-of-view PET bed positions, in order to limit overall patient radiation exposure for the CT portion of the PET/CT, and with 10 minutes of PET imaging for each field-of-view PET bed position). All patients fasted for a minimum of 6 hours before undergoing the same-day preoperative diagnostic whole-body ^18^F-FDG PET/CT scan. Only a single intravenous dose of ^18^F-FDG was used on the day of surgery, and was attempted to be administered approximately 75 minutes prior to the planned time of the same-day preoperative diagnostic whole-body ^18^F-FDG PET/CT scan, which was performed within the time frame recognized by the Society of Nuclear Medicine for ^18^F-FDG PET/CT image acquisition [63]. The ^18^F-FDG PET/CT images were acquired on one of three clinical diagnostic scanners: (1) Siemens Biograph 16 (Siemens, Knoxville, Tennessee); (2) Phillips Gemini TF (Philips, Amsterdam, Netherlands); and (3) Siemens Biograph mCT (Siemens, Knoxville, Tennessee). Only those patients with ^18^F-FDG-avid lesions seen on both same-day preoperative diagnostic whole-body ^18^F-FDG PET/CT scan and same-day postoperative diagnostic limited field-of-view ^18^F-FDG PET/CT scan were used in the current retrospective analysis. For any individual patient, the same-day preoperative diagnostic whole-body ^18^F-FDG PET/CT scan and same-day postoperative diagnostic limited field-of-view ^18^F-FDG PET/CT scan were performed on the same clinical diagnostic scanner.

The same-day preoperative diagnostic whole-body ^18^F-FDG PET/CT images and same-day postoperative diagnostic limited field-of-view ^18^F-FDG PET/CT images were evaluated by two nuclear medicine physicians who were initially blinded to all clinical information related to each set of preoperative and postoperative ^18^F-FDG PET/CT images. The two nuclear medicine physician readers first judged the quality of the preoperative and postoperative ^18^F-FDG PET/CT images as either being of diagnostic image quality or of non-diagnostic image quality, based upon criteria that were previously reported [64]. The two readers evaluated each set of preoperative and postoperative ^18^F-FDG PET/CT images for identification of all ^18^F-FDG-avid lesions that were considered suspicious for or consistent with malignancy. The location and maximum standard uptake value (SUV_max_) of each ^18^F-FDG-avid lesion were recorded. Likewise, a corresponding background SUV_max_ was obtained either from (1) an area of tissue deemed as normal within the same organ as the ^18^F-FDG-avid lesion; (2) an area of tissue deemed as normal in a location adjacent to the ^18^F-FDG-avid lesion; or (3) within a single area of tissue deemed as normal elsewhere within the body when multiple ^18^F-FDG-avid lesions were being evaluated in an individual case. The corresponding background SUV_max_ values were taken from the same location on both the preoperative and postoperative ^18^F-FDG PET/CT scans. Finally, the two readers were given access to the operative report for each case corresponding to each preoperative and postoperative ^18^F-FDG PET/CT images data set, in order to determine which ^18^F-FDG-avid lesions had been: (1) completely surgically resected; (2) partially surgically resected or biopsied; or (3) not surgically manipulated or altered (i.e., intentionally left *in situ* within the patient at the time of the ^18^F-FDG-directed surgical procedure). The ^18^F-FDG PET/CT images were all analyzed/processed on a Philips Extended Brilliance Work Station (Philips, Amsterdam, Netherlands).

All continuous variables were expressed as mean (±SD, range). The software program IBM SPSS® 21 for Windows® (SPSS, Inc., Chicago, Illinois) was used for the data analysis. All mean value comparisons for continuous variables (including the comparisons for ^18^F-FDG-avid lesion SUV_max_ values, background SUV_max_ values, and lesion-to-background SUV_max_ ratios) from the preoperative ^18^F-FDG PET/CT image group and the postoperative ^18^F-FDG PET/CT image group were performed by using the 2-tailed paired samples t-test. All categorical variable comparisons were made using 2 × 2 contingency tables that were analyzed by either the Pearson chi-square test or the Fisher exact test, when appropriate. P-values determined to be 0.05 or less were considered to be statistically significant.

## Results

### Derivation of the final ^18^F-FDG-avid lesion data set

From a total of 166 patients who gave consent to participate in one of the IRB-approved protocols, a total of 157 patients were taken to the operating room for ^18^F-FDG-directed surgery. A total of 31 of the 157 patients underwent both a same-day preoperative diagnostic whole-body ^18^F-FDG PET/CT scan and a same-day postoperative diagnostic ^18^F-FDG PET/CT scan utilizing a single same-day preoperative intravenous injection of ^18^F-FDG.

These 31 sets of preoperative and postoperative ^18^F-FDG PET/CT images were evaluated by two nuclear medicine physicians for determination of diagnostic image quality versus non-diagnostic image quality. All of the 31 preoperative ^18^F-FDG PET/CT imaging studies were determined to be of diagnostic image quality. A total of 5 of the 31 postoperative ^18^F-FDG PET/CT imaging studies were determined to be of non-diagnostic image quality. The average injection-to-scan time for these 5 postoperative ^18^F-FDG PET/CT studies with non-diagnostic image quality was of significantly longer duration, at 719 minutes (±90, 612-853), as compared to 530 minutes (±79, 413-739) for the remaining 26 postoperative ^18^F-FDG PET/CT studies with diagnostic image quality (P < 0.001), suggesting that the finding of non-diagnostic image quality on a postoperative ^18^F-FDG PET/CT scan was a direct consequence of any given postoperative ^18^F-FDG PET/CT scan being performed at the extreme outer-limit of the extended injection-to-scan acquisition time interval. No other ^18^F-FDG PET/CT imaging variables or any patient variables were significantly different for the postoperative non-diagnostic image quality group as compared to the postoperative diagnostic image quality group.

From the 26 remaining matching sets of preoperative and postoperative ^18^F-FDG PET/CT studies that were determined to be of diagnostic image quality, a total of 87 individual ^18^F-FDG-avid lesions were identified on the preoperative ^18^F-FDG PET/CT images. There were 30 ^18^F-FDG-avid lesions identified on the preoperative ^18^F-FDG PET/CT images that were completely surgical resected, 10 ^18^F-FDG-avid lesions that were partially surgically resected or biopsied, and 12 ^18^F-FDG-avid lesions were not within the field of view that was utilized on the postoperative ^18^F-FDG PET/CT images (as the postoperative ^18^F-FDG PET/CT scan was performed in a limited fashion to only to the bed of the surgical resection field). Therefore, these 52 of the original 87 individual ^18^F-FDG-avid lesions identified on the preoperative ^18^F-FDG PET/CT images were not considered for further data analysis.

The remaining 35 ^18^F-FDG-avid lesions identified on the preoperative ^18^F-FDG PET/CT images were determined to represent preoperative ^18^F-FDG-avid lesions that had not been surgically manipulated and were left *in situ* within the patient at the time of the surgical procedure, and were within the field of view on the postoperative ^18^F-FDG PET/CT images. There were 3 of these remaining 35 preoperative ^18^F-FDG-avid lesions that were not ^18^F-FDG-avid on the postoperative ^18^F-FDG PET/CT images. Of the 3 preoperative ^18^F-FDG-avid lesions not found to be ^18^F-FDG-avid on the postoperative ^18^F-FDG PET/CT images, 2 preoperative ^18^F-FDG-avid lesions were located within the bilateral tonsils in a patient who was later confirmed to have recurrent thyroid cancer within the mediastinum, but without any evidence of metastatic spread to the tonsils. These 2 areas of preoperative mild focal ^18^F-FDG-avidity seen within the bilateral palatine tonsils (SUV_max_ 4.3 on the left and 4.0 on the right), but not found to be ^18^F-FDG-avid on the postoperative PET/CT images, were determined to be secondary to nonmalignant inflammation, a well-known pitfall of diagnostic ^18^F-FDG PET/CT imaging of the tonsillar region. The third preoperative ^18^F-FDG-avid lesion was located within the stomach region of a patient with diffuse metastatic serous ovarian cancer. This area of preoperative focal ^18^F-FDG-avidity seen within the stomach region (SUV_max_ 10.0), but not found to be ^18^F-FDG-avid on the postoperative ^18^F-FDG PET/CT images, has not been further evaluated to date secondary to the lack of performance of any subsequent follow-up diagnostic ^18^F-FDG PET/CT imaging. As such, these 3 ^18^F-FDG-avid lesions were not considered for further data analysis. In the end, a total of 32 of the original 87 individual ^18^F-FDG-avid lesions identified on the preoperative ^18^F-FDG PET/CT images were considered as the final ^18^F-FDG-avid lesion data set for the current retrospective data analysis comparing the preoperative and postoperative ^18^F-FDG PET/CT images. The region of the body in which these 32 ^18^F-FDG-avid lesions were located was designated as the thorax for 12 lesions, abdomen/pelvis for 11 lesions, neck for 5 lesions, and axilla for 4 lesions.

### Patient variables

The 32 ^18^F-FDG-avid lesions, constituting the final ^18^F-FDG-avid lesion data set, originated from a total 7 patients (5 females and 2 males) from among the initial group of 31 patients who had undergone both a same-day preoperative diagnostic whole-body ^18^F-FDG PET/CT scan and a same-day postoperative diagnostic ^18^F-FDG PET/CT scan. For those 7 patients, the mean patient age was 65 (±12, 43-80) years, the mean patient weight was 80.3 (±28.1, 56.7-136.1) kilograms, the mean preoperative blood glucose level of 103 (±15, 82-121) milligrams/deciliter, and the mean intravenous ^18^F-FDG dose used on the day of surgery was 559 (±104, 437-755) megabecquerels. A histologic diagnosis of malignancy was known to be lymphoma in 3 cases, colorectal carcinoma in 2, breast carcinoma in 1, and ovarian carcinoma in 1.

### P**reoperative and postoperative **^**18**^**F-FDG PET/CT scan variables for the 32 **^**18**^**F-FDG-avid lesions and corresponding background areas**

For the 32 ^18^F-FDG-avid lesions, the mean injection-to-scan times of the preoperative and postoperative ^18^F-FDG PET/CT scans were 73 (±3, 70-78) minutes and 530 (±79, 413-739) minutes, respectively (P < 0.001). The preoperative and postoperative mean ^18^F-FDG-avid lesion SUV_max_ values were 7.7 (±4.0, 3.6-19.5) and 11.3 (±6.0, 4.1-29.2), respectively (P < 0.001). The preoperative and postoperative mean background SUV_max_ values were 2.3 (±0.6, 1.0-3.2) and 2.1 (±0.6, 1.0-3.3), respectively (P = 0.017). The preoperative and postoperative mean lesion-to-background SUV_max_ ratios were 3.7 (±2.3, 1.5-9.8) and 5.8 (±3.6, 1.6-16.2), respectively, (P < 0.001) (Table 1).

**Table 1:** **Preoperative and postoperative **^**18**^**FDG PET/CT scan variables for the 32 **^**18**^**F-FDG-avid lesions and corresponding background areas**

**Variable**	**Preoperative scan value**	**Postoperative scan value**	**P-value**
**Injection-to-scan time (minutes)**	73 (±3, 70-78)	530 (±79, 413-739)	<0.001
^**18**^**F-FDG-avid lesion SUV**_**max**_	7.7 (±4.0, 3.6-19.5)	11.3 (±6.0, 4.1-29.2)	<0.001
**Background SUV**_**max**_	2.3 (±0.6, 1.0-3.2)	2.1 (±0.6, 1.0-3.3)	0.017
**Lesion-to-background SUV**_**max **_**ratio**	3.7 (±2.3, 1.5-9.8)	5.8 (±3.6, 1.6-16.2)	<0.001

All variables are expressed as mean (±SD, range).

*Abbreviations:*^*18*^*F-FDG *^18^F-fluorodeoxyglucose, *PET/CT* positron emission tomography/computed tomography, *SUV*_*max*_ maximum standard uptake value.

**Figure 1:**
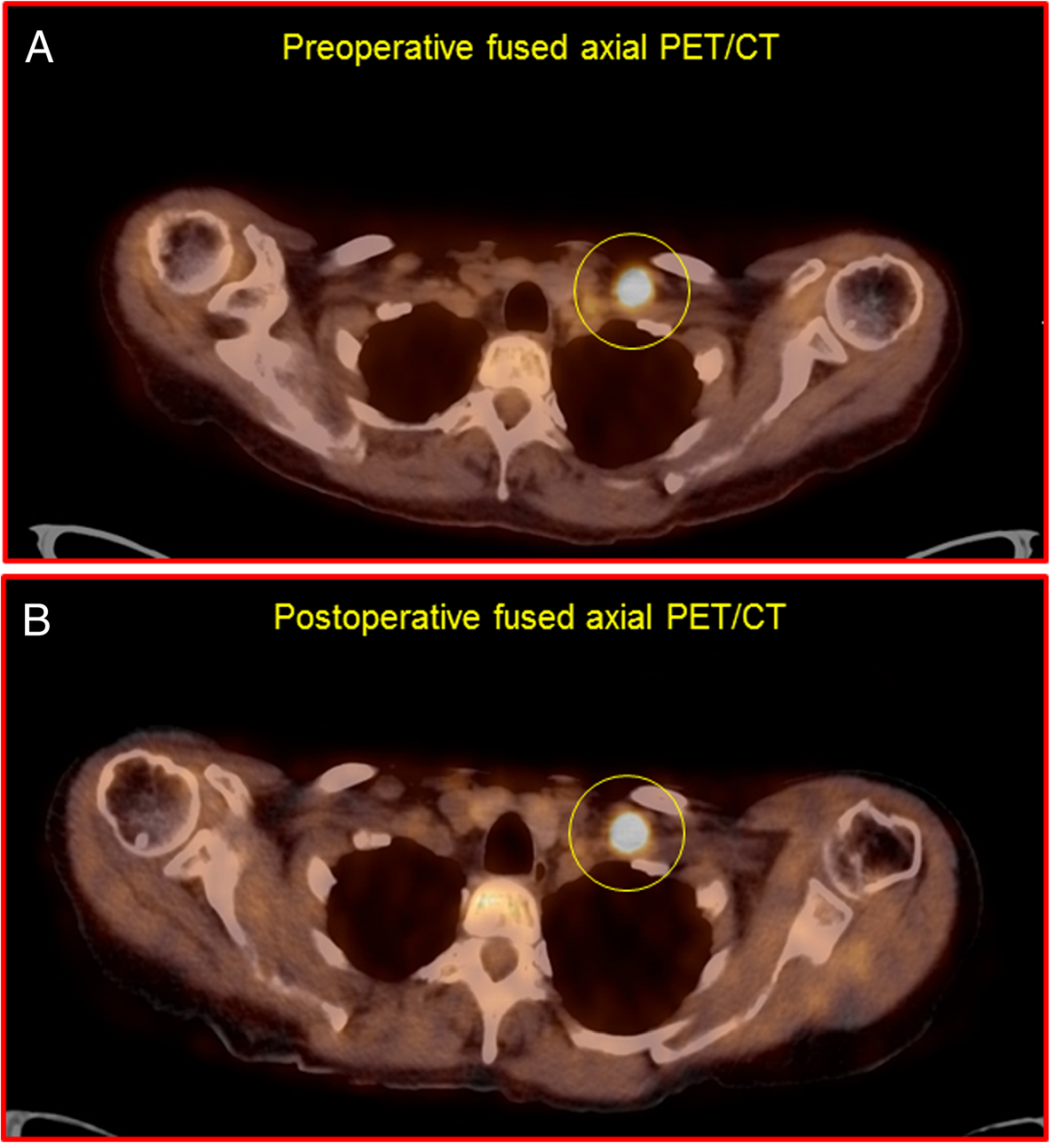
**A representative example of an **^**18**^**F-FDG-avid lesion in the left supraclavicular region (shown within the yellow circle) as seen on both same-day preoperative diagnostic whole-body **^**18**^**F-FDG PET/CT scan (panel A; SUV**_**max **_**of 16.7 at 70 minutes post-injection of 455 megabecquerels of **^**18**^**F-FDG) and same-day postoperative diagnostic limited field-of-view **^**18**^**F-FDG PET/CT scan (panel B; SUV**_**max **_**of 20.5 at 494 minutes post-injection of **^**18**^**F-FDG) in a patient with metastatic ovarian carcinoma.**

**Figure 2:**
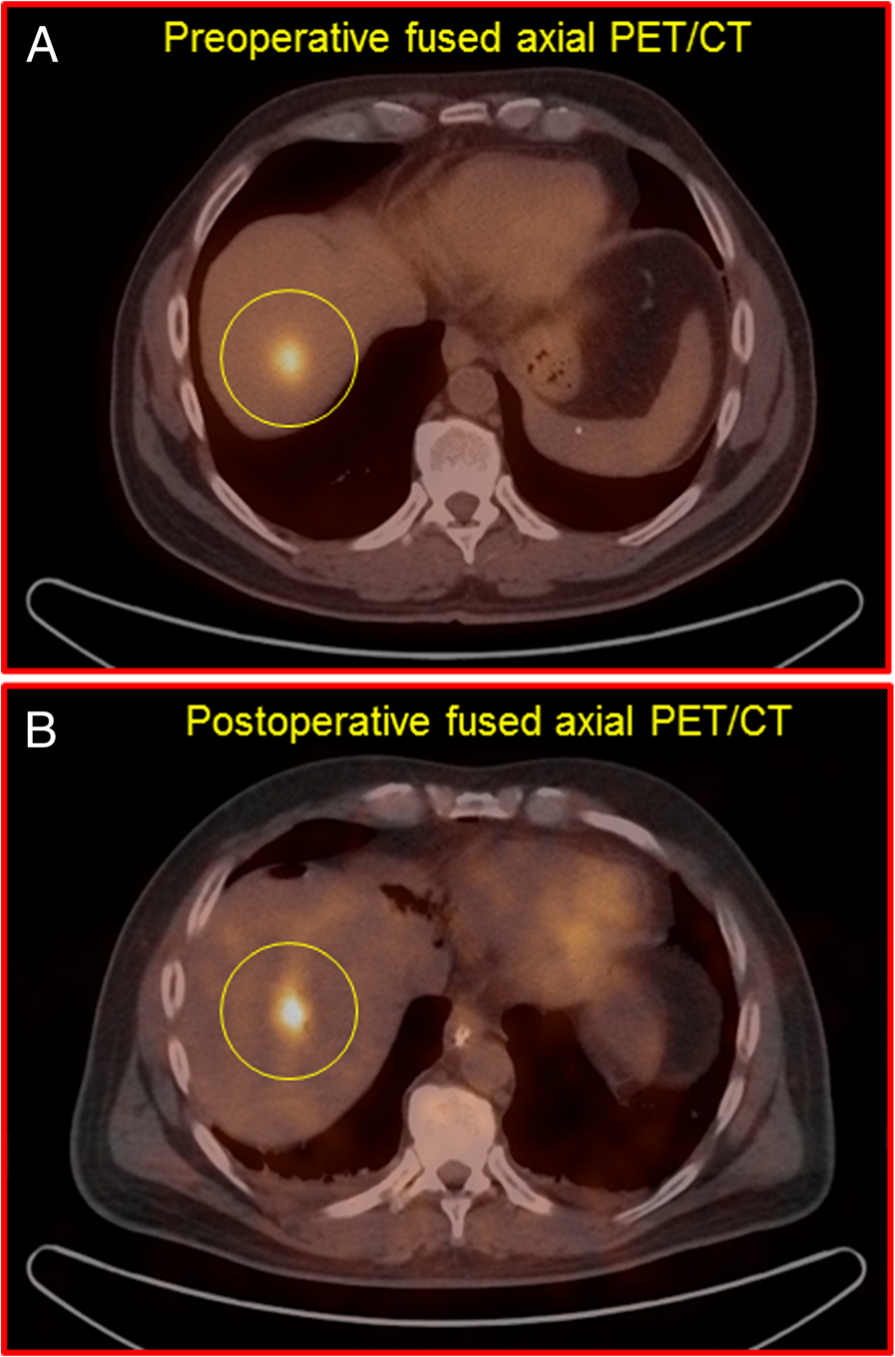
**A representative example of an **^**18**^**F-FDG-avid lesion in the right hepatic lobe of the liver (shown within the yellow circle) as seen on both same-day preoperative diagnostic whole-body **^**18**^**F-FDG PET/CT scan (panel A; SUV**_**max **_**of 8.2 at 73 minutes post-injection of 585 megabecquerels of **^**18**^**F-FDG) and same-day postoperative diagnostic limited field-of-view **^**18**^**F-FDG PET/CT scan (panel B; SUV**_**max **_**of 9.8 at 688 minutes post-injection of **^**18**^**F-FDG) in a patient with metastatic colorectal carcinoma.**

Two representative example cases of an ^18^F-FDG-avid lesion seen on both same-day preoperative diagnostic whole-body ^18^F-FDG PET/CT scan and same-day postoperative diagnostic limited field-of-view ^18^F-FDG PET/CT scan are shown in Figures 1 and 2.

Of the 32 ^18^F-FDG-avid lesions examined, only 1 ^18^F-FDG-avid lesion demonstrated a reduction in the lesion-to-background SUV_max_ ratio from the preoperative to the postoperative ^18^F-FDG PET/CT images. This particular ^18^F-FDG-avid lesion was located in the ascending colon of a patient with colorectal carcinoma, having a preoperative ^18^F-FDG-avid lesion SUV_max_ of 7.9 (with a preoperative background SUV_max_ of 1.0) and a postoperative ^18^F-FDG-avid lesion SUV_max_ of 7.5 (with a postoperative background SUV_max_ of 1.2), resulting in a change in the lesion-to-background SUV_max_ ratio of -1.7 from the preoperative to the postoperative study. Interestingly, on a subsequent follow-up diagnostic whole-body ^18^F-FDG PET/CT scan performed 9 months after ^18^F-FDG-directed surgery, the same area of this particular former ^18^F-FDG-avid lesion in the ascending colon was no longer characterized as ^18^F-FDG-avid, demonstrating a SUV_max_ of 2.1 (with a background SUV_max_ of 1.7).

For the 32 ^18^F-FDG-avid lesions, the corresponding background SUV_max_ values were taken from contralateral axillary region (n = 13), normal mediastinum (n = 10), contralateral supraclavicular region (n = 4), normal adjacent liver parenchyma (n = 2), hepatic flexure (n = 1), descending colon (n = 1), and adjacent normal spleen (n = 1).

## Discussion

The results of the current retrospective data analysis, comparing preoperative and postoperative ^18^F-FDG PET/CT imaging for 32 individual ^18^F-FDG-avid lesions (not surgically manipulated or altered during ^18^F-FDG-directed surgery, and for which all such ^18^F-FDG-avid lesions were visualized on both preoperative and postoperative ^18^F-FDG PET/CT imaging), yielded several very important observations. First, ^18^F-FDG PET/CT imaging performed at extended injection-to-scan acquisition times of up to a mean time of 530 minutes (i.e., approximately 5 half-lives for ^18^F-FDG) was able to maintain a designation of good/adequate diagnostic image quality deemed necessary for clinical interpretation. Second, the mean ^18^F-FDG-avid lesion SUV_max_ value increased significantly from preoperative to postoperative ^18^F-FDG PET/CT imaging (7.7 to 11.3; P < 0.001). Third, mean background SUV_max_ value decreased significantly from preoperative to postoperative ^18^F-FDG PET/CT imaging (2.3 to 2.1; P = 0.017). Fourth, the mean lesion-to-background SUV_max_ ratio increased significantly from preoperative to postoperative ^18^F-FDG PET/CT imaging (3.7 to 5.8; P < 0.001). These collective observations from our current analysis have potential far-reaching implications regarding the currently held premises related to ^18^F-FDG PET/CT oncologic imaging.

Multiple investigators [65-169] have evaluated the concepts of delayed phase and dual-time-point diagnostic ^18^F-FDG PET imaging approaches. In these numerous studies, attempts have been made to qualify and quantify the impact of the length of the injection-to-scan time interval on differentiating malignant processes from benign processes. As one might expect, the findings reported amongst these various investigators have been highly variable, with some supporting the use of delayed phase and dual-time-point diagnostic ^18^F-FDG PET imaging approaches [66-77,81-84,86,87,91-93,95-100,103-108,110,111,113,114,117-122,124-128,131,133,134,136,138,141,143,146,149,152,153,155,157,160-163,165,167,169], and with others not [65,78,89,90,94,101,102,109,115,116,123,129,130,132,135,137,139,140,147,148,150,151,156,158,164,166,168].

The inherent difference in intracellular glucose-6-phosphatase levels, as it relates to benign cells and tumor cells, can be used to support the notion that the delayed phase and dual-time-point diagnostic ^18^F-FDG PET/CT imaging approaches are advantageous [36,100,111,154,159,170-176]. Initially, benign cells, such as in the case of inflammatory processes, may appear hypermetabolic as they transport increased number of glucose molecules into their cytoplasm. However, the glucose is not indefinitely retained secondary to the fact that those benign cells contain normal levels of intracellular glucose-6-phosphatase, thus allowing glucose molecules to subsequently exit the cytoplasm of those cells via glucose transporter membrane proteins. On the other hand, tumor cells have decreased levels of intracellular glucose-6-phosphatase, thus allowing for a continuous accumulation of ^18^F-FDG into tumor cell over time. Therefore, methodologies that use a delayed phase in their diagnostic ^18^F-FDG PET imaging approach should allow for an expected gradual decline in intracellular ^18^F-FDG retention within initially hypermetabolic-appearing benign tissues as compared to the continued accumulation of intracellular ^18^F-FDG within malignant tissues [100,111,154,159].

Nevertheless, there are several reasons why the notion that delayed phase and dual-time-point diagnostic ^18^F-FDG PET imaging approaches are advantageous may not be so simple and clear cut. First, it is well-recognized that there can be a significant degree of overlap in the pattern of ^18^F-FDG uptake between benign tissues and various malignant tissues [154,159]. Second, there are substantial inherent variations in the methodology used in various delayed phase and dual-time-point diagnostic ^18^F-FDG PET imaging protocols from institution to institution, with great variability in the timing of the initial scan and the delayed scan, as well as a general paucity of data where the delayed scan is performed at very extended injection-to-scan acquisition time intervals after the initial time of ^18^F-FDG injection. Collectively, the vast majority of the reported series within the literature performed their delayed scan within approximately 1.5 to 2.5 hours from the initial time of ^18^F-FDG injection [65,67,70-74,79,82,83,85-93,97-100,102-104,106,107,109,110,112,113,115-127,129-135,137-143,145-169], and with far fewer series reporting their delayed scan at injection-to-scan acquisition times of approximately 3 hours or more from the initial time of ^18^F-FDG injection [66,68,69,75-78,80,81,84,94-96,101,105,108,111,114,128,136,144].

There are 5 groups of investigators, Lodge et al. in 1999 [68], Spence et al. in 2004 [81], Basu et al. in 2009 [111], Horky et al. in 2011 [136], and Prieto et al. in 2011 [144], who all performed delayed phase diagnostic ^18^F-FDG PET imaging at ultra-extended injection-to-scan acquisition time intervals, for which their clinical findings are particularly noteworthy of further discussion.

As pertaining specifically to ^18^F-FDG PET imaging for brain tumors, there have been 3 clinical series that have reported successful delayed imaging extending out to ultra-extended injection-to-scan acquisition time intervals [81,136,144]. Spence et al. reported dual-time-point diagnostic ^18^F-FDG PET imaging in various brain tumors with a median time of 5.4 hours (range of 2.9 to 9.4 hours) after ^18^F-FDG injection for the delayed scan in a series of 25 patients [81]. Prieto et al. reported dual-time-point diagnostic ^18^F-FDG PET/CT imaging in gliomas with a range of 180 to 480 minutes after ^18^F-FDG injection for the delayed scan in a series of 19 patients [144]. In both series [81,144], they reported better tumor identification and delineation, and advocated the use of delayed intervals imaging. Horky et al. reported dual-time-point diagnostic ^18^F-FDG PET imaging in patients treated with radiation for brain metastases, with delayed scans performed at a mean time of 225 minutes (range of 118 to 343 minutes) after the early scan done at 45 to 60 minutes after ^18^F-FDG injection in a series of 32 patients [136]. They found that although the early and late SUV_max_ values of the lesions alone did not differentiate residual tumor from post-radiation necrosis, the change in the lesion-to-gray matter early SUV_max_ ratio to late SUV_max_ ratio did.

Along similar lines for ^18^F-FDG PET imaging of soft tissues masses, Lodge et al. reported a series of 29 patients in which a 6-hour ^18^F-FDG PET imaging protocol was used [68]. In this protocol, a 2-hour dynamic emission data acquisition was performed after ^18^F-FDG administration, followed by 2 further 30-minute static scans, which were started at 4 hours and 6 hours after ^18^F-FDG administration. They found that the SUV value for high-grade sarcomas increased with time, reaching a peak SUV value at approximately 4 hours after initial ^18^F-FDG administration, while benign soft tissue lesions reached a maximum SUV value within approximately 30 minutes after initial ^18^F-FDG administration. They concluded that improved differentiation of high-grade sarcomas from benign soft tissue lesions was aided by SUV values derived from delayed intervals imaging.

Likewise, for ^18^F-FDG PET imaging of non-small cell lung cancer, Basu et al. reported on 3 patients in whom an 8-hour ^18^F-FDG PET imaging protocol was used [111]. In this protocol, ^18^F-FDG PET imaging was performed, starting at 5 minutes, and continuing at 1, 2, 4, 6, and 8 hours after initial ^18^F-FDG administration. They found that sites of non-small cell lung cancer showed a progressive increase in ^18^F-FDG uptake over the 8-hour course, while surrounding normal tissues demonstrated either a declining or stable pattern of ^18^F-FDG uptake with time. They concluded that delayed injection-to-scan acquisition time intervals had “implications in detecting malignant lesions with greater degree of certainty”…“due to better contrast between the abnormal site and the surrounding background”.

Of last mention, similar recommendations for the use of delayed injection-to-scan acquisition time interval imaging have been made by other investigators at somewhat less extended injection-to-scan acquisition time intervals of approximately 3 hours in breast cancer [66,105,128], cervical cancer [76,77], hepatocellular cancer [84], biliary malignancies [95], lung cancer [75,96,108], and thymic epithelial tumors [114].

The results of the previously reported series demonstrating their ability to successfully perform delayed imaging at extended injection-to-scan acquisition time intervals of approximately 3 hours or more from the initial time of ^18^F-FDG injection [66,68,69,75-78,80,81,84,94-96,101,105,108,111,114,128,136,144], as well as those demonstrating the added value to performing delayed imaging at extended injection-to-scan acquisition time intervals of approximately 3 hours or more from the initial time of ^18^F-FDG injection [66,68,75-77,81,84,95,96,105,108,111,114,128,136,144], are all highly consistent with the results of our current retrospective data analysis. It is clear that our currently presented data, demonstrating increasing ^18^F-FDG-avid lesion SUV_max_ values, decreasing background SUV_max_ values, and increasing lesion-to-background SUV_max_ ratios from preoperative to postoperative ^18^F-FDG PET/CT imaging, supports the potential utility of delayed phase and dual-time-point diagnostic ^18^F-FDG PET/CT imaging. This suggests that delayed scans performed at an appropriately selected extended injection-to-scan acquisition times can potentially minimize or alleviate the issue of overlap in the pattern of ^18^F-FDG uptake between benign tissues versus malignant tissues, as well as between background tissues versus malignant tissues. This phenomenon appears to be the temporal outcome of a resultant gradual accumulation of ^18^F-FDG within malignant tissues and continued decreased background level of ^18^F-FDG within the surrounding normal tissues, thus leading to a progressive increase in the lesion-to-background SUV_max_ ratio. A key element to this overall line of reasoning, as it relates to the proper use of ^18^F-FDG in molecular imaging, is the recognition of the negative impact of “background” issues, and “not signal”, as recently eloquently described by Frangioni [177], but which was recognized early on in the evolution of PET imaging by Hoffman and Phelps [178]. This time-dependent phenomenon observed in our current retrospective analysis is consistent with our previously reported findings regarding same-day preoperative diagnostic whole-body ^18^F-FDG PET/CT images and same-day perioperative *ex vivo* surgical specimen ^18^F-FDG PET/CT imaging, in which we observed similar trends of increased ^18^F-FDG accumulation in ^18^F-FDG-avid lesions within *ex-vivo* surgical specimens and of decreased ^18^F-FDG activity within adjacent normal tissues [37]. However, we fully acknowledge and recognize that significant further investigations are warranted to better assess this phenomenon and to formally evaluate the clinical usefulness of extended injection-to-scan acquisition time intervals in various diagnostic ^18^F-FDG PET/CT oncologic imaging applications.

Analogous to our current discussions regarding the evaluation and quantification of ^18^F-FDG-avid lesions and corresponding background tissues at these extended injection-to-scan acquisition time intervals for ^18^F-FDG PET imaging approaches, there have been two groups of investigators utilizing ^18^F-FDG-directed surgery [11,17,21], other than our own collaborative group [51], who have previously examined the equivalent question as it pertains to the impact of the length of time from injection of ^18^F-FDG to the performance of intraoperative gamma detection probing [11,17,21]. One such group [17,21] recognized that there was an increased tumor-to-background ratio of ^18^F-FDG seen during intraoperative gamma detection probing when there was a longer duration (i.e., up to 6 hours of time) from injection of the ^18^F-FDG dose to intraoperative probing. However, they did not endorse lengthening the duration from injection of the ^18^F-FDG dose to performing intraoperative gamma detection probing or to performing perioperative ^18^F-FDG PET imaging [21]. Instead, they specifically commented that lengthening the duration from injection of the ^18^F-FDG dose “might compromise image quality as a result of lower count rates” [21]. The other such group [11], as based upon the evaluation of ^18^F-FDG count rates for only three patients, concluded that intraoperative gamma detection probing was “more suitable” at 1 to 3 hours post-injection of ^18^F-FDG as compared to 6 to 7 hours post-injection of ^18^F-FDG. In both instances, these two groups of investigators fell short of recognizing the potential efficacies of extended injection-to-scan acquisition time intervals.

Although we clearly recognize that the current retrospective data analysis is based upon only 32 individual ^18^F-FDG-avid lesions, the potential significance of our current collective observations is far-reaching for ^18^F-FDG PET/CT oncologic imaging. While the possibility of ultra-extended injection-to-scan acquisition time intervals of up to approximately 5 half-lives for ^18^F-FDG was first alluded to in the dose uptake ratio simulation studies by Hamberg et al. in 1994 [179] and was later clinically examined by Lodge et al. in 1999 [68], Spence et al. in 2004 [81], Basu et al. in 2009 [111], Horky et al. [136], and Prieto et al. in 2011 [144], its potential future impact has not previously been fully realized within the nuclear medicine or surgical literature. The ability to maintain good/adequate diagnostic image quality for ^18^F-FDG PET/CT imaging at extended injection-to-scan acquisition time intervals of up to approximately 5 half-lives and the resultant time-dependent increase in the observed ^18^F-FDG-avid lesion SUV_max_ values, decrease in the observed background SUV_max_ values, and increase in the lesion-to-background SUV_max_ ratios allow for and justify the more widespread and integrated, real-time use of diagnostic ^18^F-FDG PET/CT imaging in conjunction with ^18^F-FDG-directed interventional radiology biopsy procedures and ablation procedures, as well as with ^18^F-FDG-directed surgical procedures. Such integrated, real-time utilities for diagnostic ^18^F-FDG PET/CT imaging would facilitate periprocedural verification of appropriate tissue targeting during ^18^F-FDG-directed interventional radiology biopsy procedures and ablation procedures and for perioperative verification of appropriate tissue targeting and completeness of resection during ^18^F-FDG-directed surgical procedures. Furthermore, these resultant time-dependent observations could have far-reaching impact on potentially re-shaping future thinking regarding what represents the “most optimal” injection-to-scan acquisition time interval for all routine diagnostic ^18^F-FDG PET/CT oncologic imaging, as the current procedure guideline for tumor imaging with ^18^F-FDG PET/CT, as published by the Society of Nuclear Medicine, simply states that “emission images should be obtained at least 45 minutes after radiopharmaceutical injection” [63].

## Conclusions

Our current retrospective data analysis demonstrates that ^18^F-FDG PET/CT oncologic imaging can be successfully performed at extended injection-to-scan acquisition time intervals of up to approximately 5 half-lives for ^18^F-FDG while maintaining good/adequate diagnostic image quality. The resultant increased ^18^F-FDG-avid lesion SUV_max_ values, decreased background SUV_max_ values, and increased lesion-to-background SUV_max_ ratios seen from preoperative to postoperative ^18^F-FDG PET/CT imaging have great potential for allowing for the integrated, real-time use of ^18^F-FDG PET/CT imaging in conjunction with ^18^F-FDG-directed interventional radiology biopsy and ablation procedures and ^18^F-FDG-directed surgical procedures, as well as have far-reaching impact on potentially re-shaping future thinking regarding the “most optimal” injection-to-scan acquisition time interval for all routine diagnostic ^18^F-FDG PET/CT oncologic imaging. In these regards, we fully acknowledge and recognize the need for further investigations to better assess and formally evaluate the clinical utility of extended injection-to-scan acquisition time intervals in various diagnostic ^18^F-FDG PET/CT oncologic imaging applications.

## Competing interests

All the authors declare that they have no competing interests to report.

## Authors’ contributions

SPP was responsible for the overall study design, data collection, data organization, data analysis/interpretation, writing of all drafts of the manuscript, and has approved final version of the submitted manuscript. DAM was involved in study design, data collection, data organization, data analysis/interpretation, writing portions of the manuscript, and has approved final version of the submitted manuscript. SMS was involved in data organization, data analysis, and has approved final version of the submitted manuscript. EWM was involved in discussion about study design, data analysis/interpretation, critiquing drafts of the manuscript, and has approved final version of the submitted manuscript. NCH was involved in study design, discussion about data analysis/interpretation, editing portions of the manuscript, and has approved final version of the submitted manuscript.

## Pre-publication history

The pre-publication history for this paper can be accessed here:

http://www.biomedcentral.com/1471-2407/14/453/prepub
